# Supplementary material for: Prenatal SAMe Treatment Changes via Epigenetic Mechanism/s USVs in Young Mice and Hippocampal Monoamines Turnover at Adulthood in a Mouse Model of Social Hierarchy and Depression
Source: Int J Mol Sci. 2023 Jun 27;24(13):10721. doi: 10.3390/ijms241310721 (PMC10361211; doi:10.3390/ijms241310721)
Supplement: Supplementary file 1 [file ijms-24-10721-s001.zip › ijms-2438223-supplementary.pptx]

## Slide 1
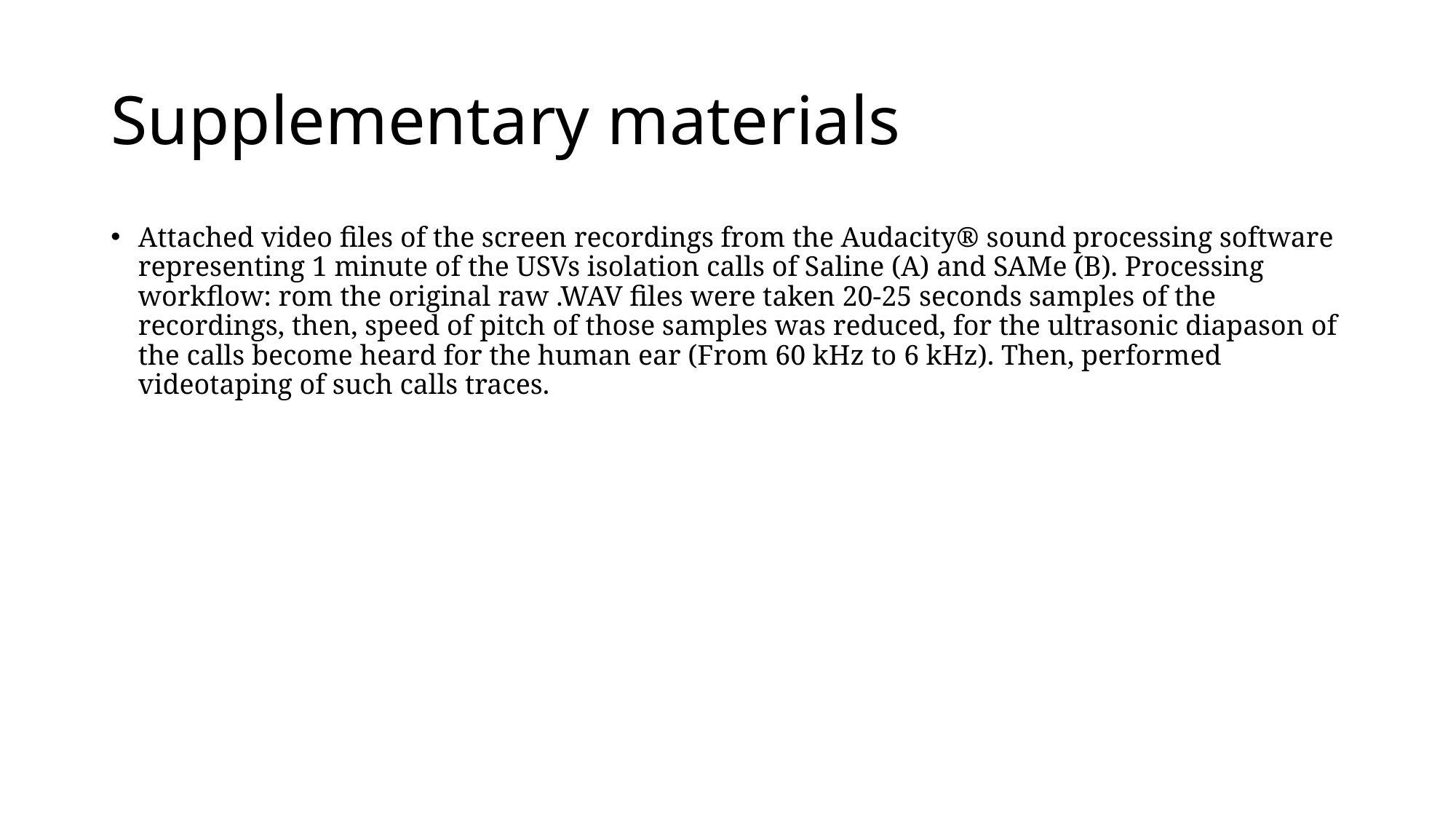

# Supplementary materials
Attached video files of the screen recordings from the Audacity® sound processing software representing 1 minute of the USVs isolation calls of Saline (A) and SAMe (B). Processing workflow: rom the original raw .WAV files were taken 20-25 seconds samples of the recordings, then, speed of pitch of those samples was reduced, for the ultrasonic diapason of the calls become heard for the human ear (From 60 kHz to 6 kHz). Then, performed videotaping of such calls traces.

## Slide 2
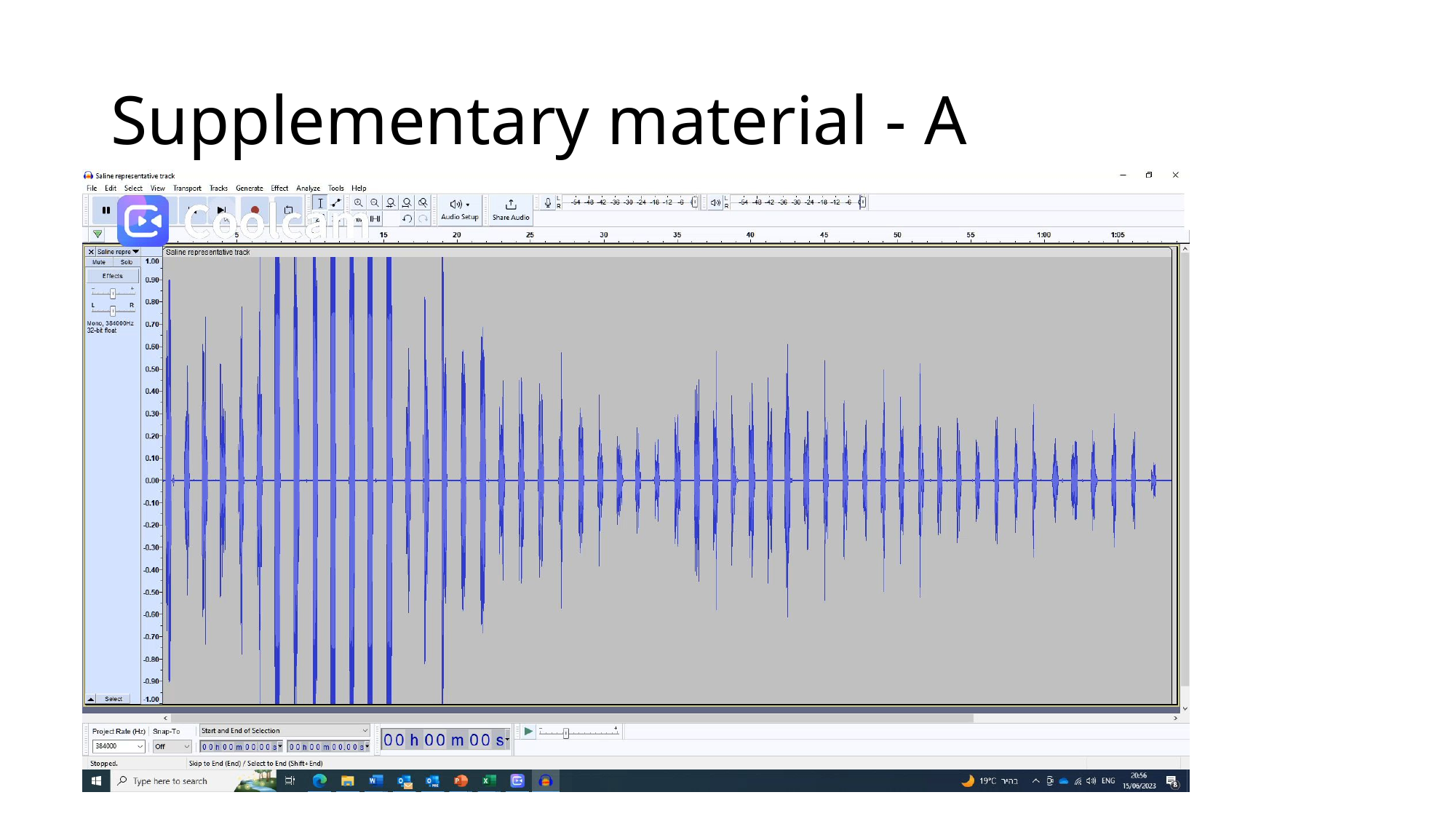

# Supplementary material - A

## Slide 3
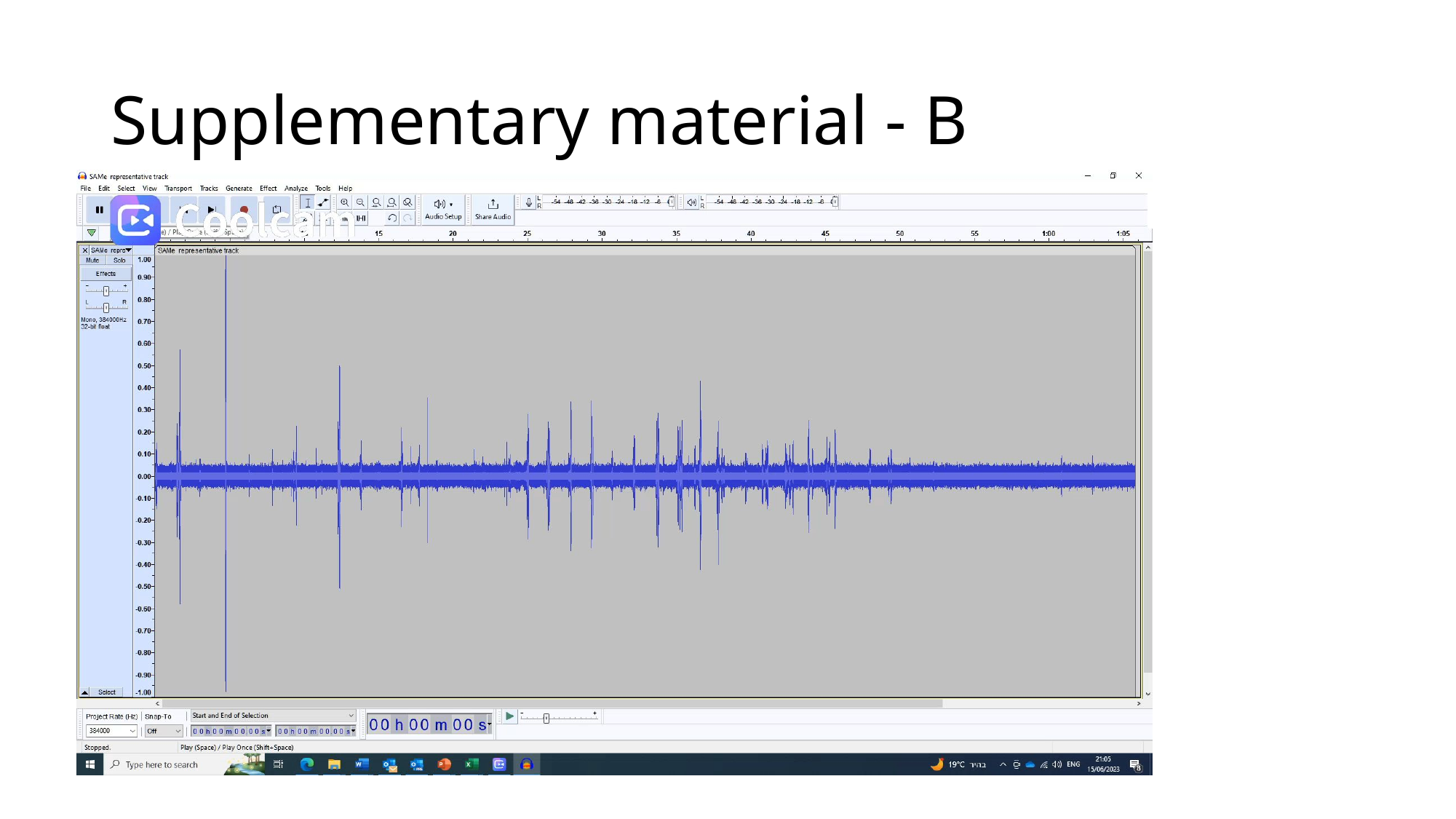

# Supplementary material - B
